# Diversity and Quantitative Detection of Clade I Type *nosZ* Denitrifiers in the Arabian Sea Oxygen Minimum Zone

**DOI:** 10.1264/jsme2.ME22056

**Published:** 2023-01-26

**Authors:** Mandar Bandekar, Nagappa Ramaiah, Seyieleno C. Seleyi, Delcy R. Nazareth, Jukka Kekäläinen

**Affiliations:** 1 Biological Oceanography Division, CSIR-National Institute of Oceanography, Dona Paula, Goa, 403004; 2 Department of Environmental and Biological Sciences, University of Eastern Finland, Joensuu Finland

**Keywords:** Arabian Sea, oxygen minimum zone, *nosZ* gene, quantitative PCR, diversity

## Abstract

A significant amount of nitrous oxide (N_2_O) is effluxed into the atmosphere as a result of marine denitrification in the Arabian Sea (AS) oxygen minimum zone (OMZ). An assessment of temporal variations in the diversity and abundance of *nosZ* denitrifiers was performed to establish the relative importance of these bacteria in denitrification. Sampling was conducted at the Arabian Sea Time Series (ASTS) location and a quantitative PCR (qPCR) ana­lysis was performed. We detected a high abundance of the *nosZ* gene at core OMZ depths (250‍ ‍m and 500 m), indicating the occurrence of denitrification in the AS-OMZ. The maximum abundance of the *nosZ* gene was observed during the Spring Intermonsoon (SIM) at 250‍ ‍m (1.32×10^6^ copies L^–1^) and 500‍ ‍m (1.50×10^6^ copies L^–1^). Sequencing ana­lysis showed that *nosZ* denitrifiers belonged to the classes *Alpha-*, *Beta-*, and *Gammaproteobacteria*. Taxonomic ana­lysis revealed that most OTUs were affiliated with *Pseudomonas*, *Rhodopseudomonas*, and *Bradyrhizobium*. Diversity indices and richness estimators confirmed a higher diversity of *nosZ* denitrifiers at 250‍ ‍m than at 500‍ ‍m during all three seasons. The present results also indicated that dissolved oxygen (DO) and total organic carbon (TOC) are critical factors influencing the diversity and abundance of the *nosZ*-denitrifying bacterial community.

By contributing up to 40% of oceanic nitrogen loss (Codispoti, 2007), predominantly due to the biogeochemical processes occurring in the oxygen minimum zone (OMZ) (Stramma *et al.*, 2008; Ulloa *et al.*, 2008), oceanic denitrification markedly affects the global nitrogen cycle. Denitrification is a large source of nitrous oxide (N_2_O, greenhouse gas), which contributes to climate change and ozone destruction (Beaulieu *et al.*, 2011). Approximately 50% of the annual emissions of ocean N_2_O are from OMZs located in the Arabian Sea (AS), Eastern Tropical South Pacific (ETSP), and Eastern Tropical North Pacific (ETNP) (Naqvi *et al.*, 2005). In OMZs, the reduction of nitrate increases the concentration of nitrite (NO_2_^–^), which is further reduced to gaseous N_2_O and nitrogen (N_2_) largely through heterotrophic microbe-mediated denitrification (Codispoti and Christensen, 1985).

The AS-OMZ is recognized as the largest suboxic region of extreme upwelling and high productivity (Wyrtki, 1973) with the low exchange of intermediary waters (150–1,000‍ ‍m) and is responsible for the greatest marine nitrogen loss (20%) (Codispoti *et al.*, 2001). Denitrification dominates the biogeochemistry of the oxygen-depleted water column of the AS and this process occurs through the diverse assemblage of heterotrophic microbes (Zumft, 1997). High abundance and disparate groups of denitrifying bacteria are present in the AS-OMZ (Jayakumar *et al.*, 2004). Not all denitrifying bacteria convert nitrous oxide to N_2_; some only produce N_2_ in the complete absence of free oxygen. Nevertheless, they undergo partial denitrification under suboxic conditions and release N_2_O (Takaya *et al.*, 2003). Therefore, denitrification is a potential source of nitrous oxide and a critical process in the AS-OMZ, which is continuously expanding

Nitrous oxide reduction is the final step in the denitrification pathway, and the only known process for the utilization of N_2_O by microbial communities is by nitrous oxide reductase (*nosZ*; Hein and Simon, 2019), a copper-containing enzyme found in all denitrifiers capable of reducing nitrate (NO_3_^–^). The *nosZ* gene has two distinct clades, Clade I (typical) and Clade II (atypical). Nitrous oxide genes associated with conventional denitrifiers are grouped as Clade I *nosZ* genes.

However, the *nosZ* gene is not always affiliated with denitrifying microbes; the recent discovery of novel *nosZ* sequences in non-denitrifying bacterial groups (Sanford *et al.*, 2012; Jones *et al.*, 2013) showed that they possess Clade II (atypical) *nosZ* genes. Clade II *nosZ* genes lack one or more denitrification genes and are known as incomplete denitrifiers (Sanford *et al.*, 2012).

The climatic impact of marine nitrous oxide emissions due to deoxygenation have led to appeals for a more detailed understanding of the biological sink and sources of this gas in the ocean. The key to assessing the budget of nitrous oxide in OMZs is clarifying the distribution and abundance of microorganisms involved in the production and consumption of N_2_O. In the present study, we investigated the diversity and abundance of denitrifying bacteria from suboxic waters of the Arabian Sea Time Series (ASTS, 17°0.126′ N, 67°59.772′ E) location. We targeted the *nosZ* gene from DNA extracts to assess community and temporal variations. Furthermore, season- and depth-wise differences in the distribution of these bacteria were examined using quantitative PCR (qPCR). This information is critical for recognizing the responses of denitrifiers to changing environmental factors. Additionally, the identification of these bacteria will provide insights into the influence of *nosZ* denitrifiers on nitrogen transformation and fluxes.

## Materials and Methods

### Water sampling

Water samples were collected from the ASTS location using Niskin bottles on a CTD rosette from the surface (5 m), deep chlo­rophyll maxima (DCM) (~43–50 m), core OMZ depths (250 and 500 m), and 1,000‍ ‍m during three seasons *i.e.* the Spring intermonsoon (SIM), Fall intermonsoon (FIM), and Northeast monsoon (NEM). Water was processed through sterivex filters (0.22‍ ‍μm; Millipore), filled with 1.7‍ ‍mL of storage buffer (50 Mm, Tris pH 8.3, 40‍ ‍mM EDTA, and 0.75 M sucrose) and stored at –80°C until DNA extraction.

### Measurements of physicochemical parameters

Physicochemical parameters (depth, temperature, salinity, and pH) in every water sample were measured using different sensors mounted onto the CTD rosette. The standard Winkler titration method (Carpenter, 1965) was used to measure dissolved oxygen (DO). Nutrient (ammonia, nitrate, nitrite, phosphate, and silicate) concentrations were assessed according to previously reported methods (Grasshoff *et al.*, 1983).

### DNA extraction and PCR amplification

Nucleic acid extraction from water samples was performed according to standard methods (Ferrari and Hollibaugh, 1999). The amplification of DNA samples was conducted using the primer set nosZ1F (5′-WCSYTGTTCMTCGACAGCCAG-3′) and nosZ1R (5′-ATGTCGATCARCTGVKCRTTYTC-3′) (Henry *et al.*, 2006). The primers nosZ1F and nosZ1R targeted Clade I genes; Clade II genes were not amplified separately. PCR was performed using a Thermocycler machine (Applied Biosystem) following temperature conditions of 95°C for 5‍ ‍min, for initial denaturation, 30 cycles at 95°C for 30‍ ‍s, annealing at 60°C for 45‍ ‍s, and a final extension step at 72°C for 90 s. A negative control (PCR mix and primers) was used in each PCR reaction and amplification was confirmed by agarose gel (1%) electrophoresis.

### Cloning and sequencing

NosZ gene amplicons were purified, cloned into the PGEM-T Easy Vector (Promega), transformed inside high efficiency JM109 cells, and grown overnight at 37°C on LB/X-gal/IPTG plates. A minimum of 40 clones were selected per plate for colony PCR. Temperature conditions for colony PCR were as follows: an initial denaturation step at 94°C for 10‍ ‍min, followed by 30 cycles at 94°C for 1‍ ‍min, annealing at 55°C for 1‍ ‍min with an elongation step at 72°C for 1‍ ‍min, and a final extension at 72°C for 10‍ ‍mins. PCR products were purified, measured, and sequenced using 15–50‍ ‍ng of amplicons, adding 10 pmol each of the nosZ1F and nosZ1R primers in an ABI 3130 Genetic Analyzer (Applied Biosystems). Temperature conditions for sequencing were an initial denaturation at 96°C for 1‍ ‍min, 30 cycles at 96°C for 10‍ ‍s, annealing at 60°C for 45‍ ‍s, elongation at 60°C for 4‍ ‍mins, and a final extension at 60°C for 1‍ ‍min.

### Sequence ana­lysis

*NosZ* gene sequences were assembled using DNA Baser sequence assembly software version 2. VecScreen was used to eliminate vector contamination. Non-chimeric consensus sequences without vector and primer residues were submitted to the National Center for Biotechnology Information (NCBI) database to obtain accession numbers and were used in additional ana­lyses. Sequences were classified using 1,000 pseudo-bootstrap replications at a bootstrap value of 80% (standard error of only 1.3%). Alignments were trimmed using Gblocks (Castresana, 2000). Clone sequences were compared with the NCBI database and assigned to a phylum if their identity was more than 95%.

### OTU assessment and phylogenetic tree

Sequences (*nosZ* gene) were assigned to operational taxonomic units (OTUs) by the average neighbor rule (Schloss and Westcott, 2011) using MOTHUR. Sequences obtained from all seasons from depths of 250 and 500‍ ‍m were grouped as nosZ250m and nosZ500m, respectively, and all *nosZ* sequences obtained from 250 and 500‍ ‍m were combined as CnosZ. OTUs were obtained at a sequence similarity of 95%, and representative sequences for each OTU of nosZ250m, nosZ500m, CnosZ, and top-hit nucleotide sequences from cultivated known strains from the NCBI database were used to build the phylogenetic tree (MEGA 6.0) with 1,000 replicate bootstrap ana­lyses.

### qPCR assay

Quantification of the *nosZ* gene was performed using the ABI 7500 Real-Time PCR system (Applied Biosystems). A plasmid carrying the *nosZ* gene fragment was cloned using the pCR4-TOPO vector and confirmed by sequencing. Ten-fold serial dilutions of a known copy number of plasmid DNA were subjected to a qPCR assay in triplicate to generate a standard curve and calculate the qPCR efficiency of the *nosZ* gene. A standard curve was generated by plotting threshold cycle values versus log10 of gene copy numbers. The slope, y-intercept, and coefficient of determination (r2) were assessed. The efficiency of amplification (E) was calculated using the following equation: E=–1+10^(–1/slope)^.

The abundance of the *nosZ* gene was quantified in triplicate. Each reaction contained a mixture of DNA (4‍ ‍μL), the primer pair nosZ1F/nosZ1R (0.5‍ ‍μL), and 5× qARTA Green qPCR Mix (12.5‍ ‍μL). PCR cycles were performed according to the standard protocol (Levy-Booth and Winder, 2010). The copy number of the target gene was calculated directly against the standard curve. The negative control had higher Ct values (~9 cycles) than the most diluted plasmid containing the target gene. Additionally, qPCR products were cloned and sequenced to confirm the identity of the gene. A post-amplification melting curve ana­lysis showed that there was no target gene contamination in the reagents.

### Statistical ana­lysis

Spearman’s correlation coefficient was used to evaluate the relationships between physicochemical parameters (DO, TOC, NO_2_^–^ NO_3_^–^, and NH_4_^+^) and gene copy numbers for every individual season. One-way ana­lysis of variance (ANOVA) was performed for each physicochemical parameter, OTU, and copy number. Paired differences between depths within each season were tested using Tukey’s post hoc tests. Principal component ana­lysis (PCA) with varimax rotations for the above-described physicochemical parameters was performed to reduce the number of inter-correlated variables. Multiple regression ana­lyses were conducted to investigate the relationships between OTUs, copy numbers (dependent variables), and principal component scores (predictor variables) obtained from PCA. Statistical ana­lyses were conducted using IBM^©^ SPSS 23.0. Each result was shown as the mean±standard deviation (SD).

Rarefaction curve, diversity indices (Shannon’s and Simpson’s), and richness estimators (Chao 1 and ACE) were evaluated using MOTHUR. Non-parametric richness estimators were used to extrapolate the total richness of clone libraries from the observed number of OTUs. Diversity and richness estimators were calculated for individual clone libraries.

### NCBI Accession numbers

NCBI accession numbers for the 171 *nosZ* gene sequences obtained in the present study are KX784867 to KX784885, KX911214 to KX911243, KY065372 to KY065445, and KY100043 to KY100090.

## Results

### Hydrography

**Fig. 1.**
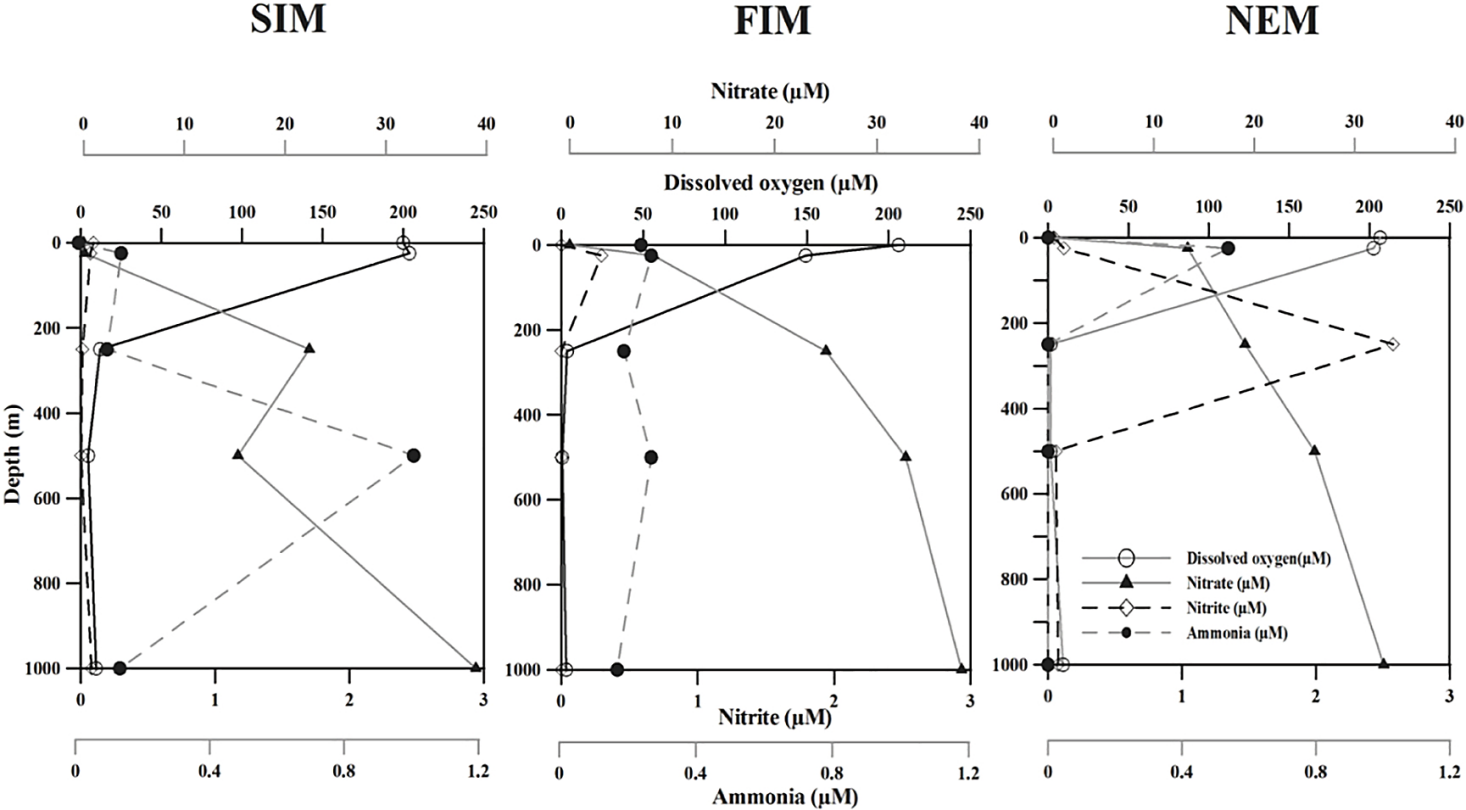
Vertical distribution of concentrations of dissolved oxygen (DO), nitrate (NO_3_^–^), nitrite (NO_2_^–^), and ammonia (NH_4_^+^) during the spring intermonsoon (SIM), fall intermonsoon (FIM), and northeast monsoon (NEM) at the Arabian Sea Time Series (ASTS) location.

Temperature, salinity, pH, and total organic carbon (TOC) were consistent, while the concentrations of DO and nutrients varied at core OMZ depths (Table 1). The average DO concentration decreased from 15.73‍ ‍μmol L^–1^ at 250‍ ‍m to 5.85‍ ‍μmol L^–1^ at 500 m. The concentration of nitrate was higher at core OMZ depths during all three seasons and ranged between 15.33 to 32.79‍ ‍μmol L^–1^. The highest concentrations of nitrite (2.57‍ ‍μmol L^–1^) and ammonia (NH_4_^+^ 1.01‍ ‍μmol L^–1^) were noted at 250 and 500‍ ‍m during the NEM and SIM, respectively. The concentrations of DO, NO_2_^–^ NO_3_^–^, and NH_4_^+^ during all three seasons are shown in Fig. 1.

### Phylogenetic ana­lyses

Evolutionary differences between OTUs and representative sequences were assessed by phylogenetic ana­lyses. PCR amplification of the *nosZ* gene (259-bp amplification product) was only positive for samples collected at 250 and 500‍ ‍m in all seasons. Among the 210 clones sequenced, 171 non-chimeric *nosZ* gene sequences were obtained and the following clone libraries were built: SIM-250, SIM-500, FIM-250, FIM-500, NEM-250, and NEM-500.

### Phylogeny of *nosZ* denitrifiers

The sequences of *nosZ* clones showed 80–93% identity with one another and 77–88% similarity to sequences in GeneBank. All OTUs obtained in the present study matched the sequences of the phylum Proteobacteria in the NCBI database. Taxonomic ana­lyses reveals that most *nosZ* OTUs were associated with *Pseudomonas*, *Rhodopseudomonas*, *Bradyrhizobium*, and *Alphaproteobacteria*. A small percentage of bacteria was affiliated with *Azospirillum*, *Achromobacter*, *Cupriavidus*, *Nisaea*, *Thalassobaculum*, *Sinorhizobium*, *Herbaspirillum*, *Burkholderia*, and *Alcaligenes*. The phylogenetic ana­lysis showed that OTUs in the present study closely matched the environmental sequences obtained from the suboxic zone of the AS, deep-sea waters of the Mediterranean Sea, the crop soil and wetland sediment of Mexico, terrestrial subsurface sediments, a marine aquaculture biofilter, and paddy soil.

Among the 87 clones sequenced from nosZ250m, 17 OTUs were obtained (Fig. S1), the majority of which were affiliated with the genera *Rhodopseudomonas* (18%, 3 OTUs) and *Pseudomonas* (18%, 3 OTUs). *Azospirillum* (2‍ ‍OTUs), *Bradyrhizobium* (2 OTUs), *Achromobacter* (2 OTUs), and *Cupriavidus* (2 OTUs) each contributed 12% of the total number of OTUs. Bacterial sequences belonging to the genera *Nisaea* (1 OTU), *Thalassobaculum* (1 OTU), and class *Alphaproteobacteria* (1 OTU) formed OTUs with a smaller number of clones.

Thirteen OTUs were generated from nosZ500m (84 sequences) (Fig. S2), the majority of which belonged to the genera *Bradyrhizobium* (15%, 2 OTUs), *Pseudomonas* (15%, 2 OTUs), and *Alphaproteobacteria* (15%, 2 OTUs). *Thalassobaculum* (1 OTU), *Rhodopseudomonas* (1 OTU), *Sinorhizobium* (1 OTU), *Azospirillum* (1 OTU), *Herbaspirillum* (1 OTU), *Burkholderia* (1 OTU), and *Alcaligenes* (1 OTU) were the other minor OTUs present.

**Fig. 2.**
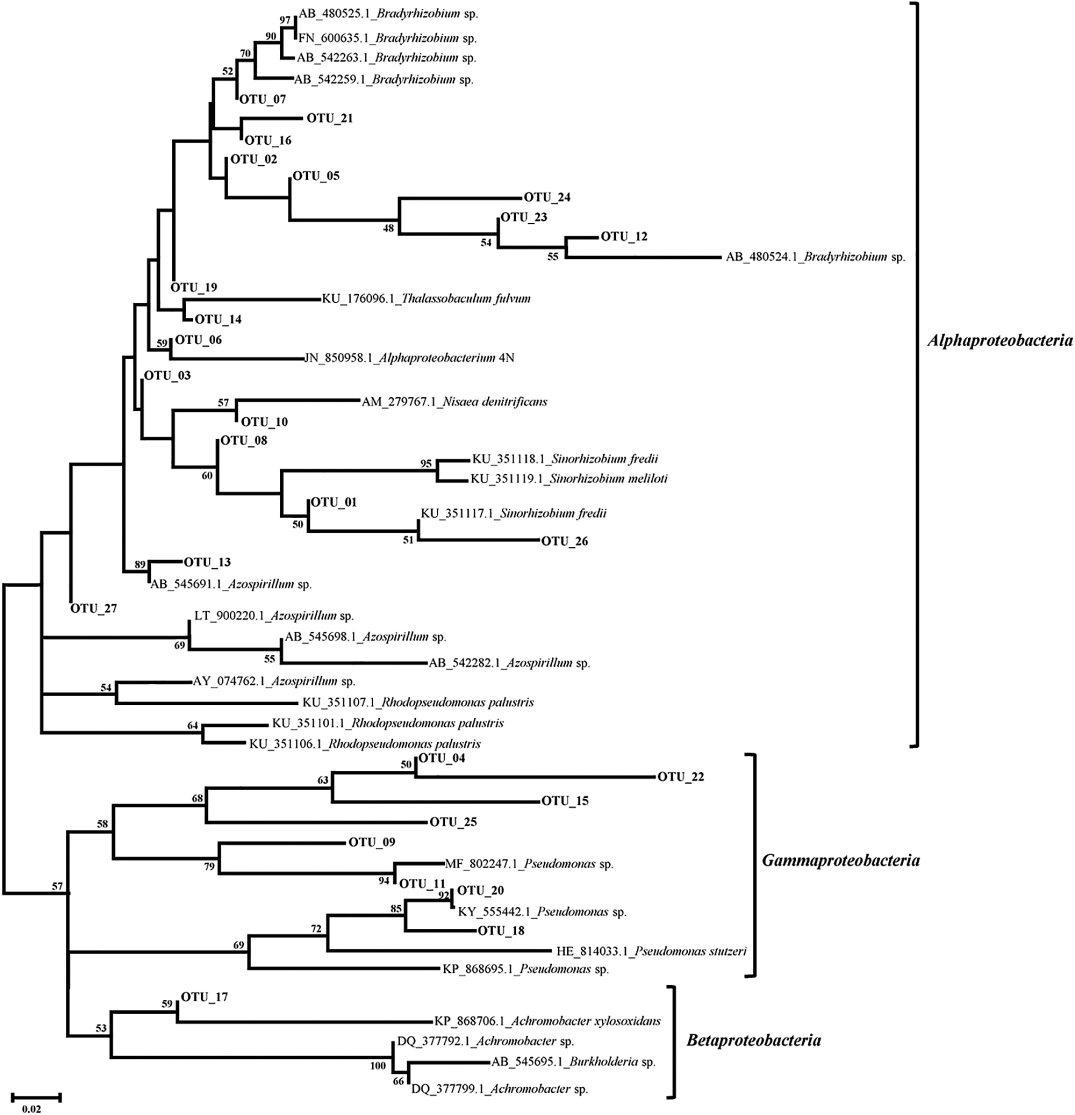
Phylogenetic tree constructed using the neighbor-joining method of *nosZ* gene OTU sequences obtained from CnosZ500m at the ASTS location. Sequences in bold are from the present study.

**Fig. 3.**
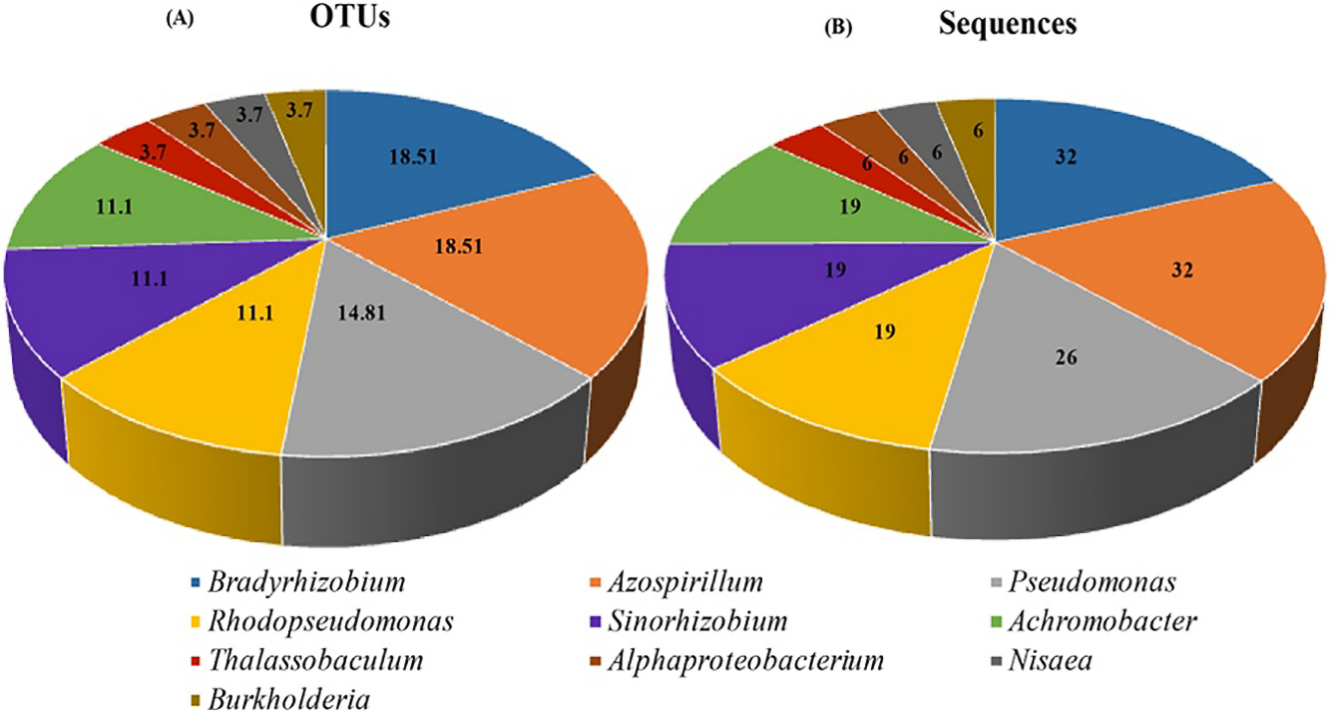
Percentages of the relative abundance and sequence numbers of nosZ gene OTU sequences obtained from CnosZ at the Arabian Sea Time Series (ASTS) location.

Among the 171 clones sequenced from core OMZ depths, 27 OTUs were generated, which were affiliated with the classes *Alpha-*, *Beta-*, and *Gammaproteobacteria* (Fig. 2). The majority of OTUs were affiliated with the genera *Bradyrhizobium* (5 OTUs, 18.5%, 32 sequences), *Azospirillum* (5 OTUs, 18.5%, 32 sequences), and *Pseudomonas* (4 OTUs, 14.8%, 26 sequences). Three OTUs (11.1%) each were associated with *Rhodopseudomonas* (19 sequences), *Sinorhizobium* (19 sequences), and *Achromobacter* (19 sequences). One OTU (6 sequences) each was affiliated with the genera *Thalassobaculum*, *Nisaea*, and *Burkholderia* and *the* class *Alphaproteobacteria* (Fig. 3).

### Season- and depth-wise distribution of *nosZ* denitrifiers

**Fig. 4.**
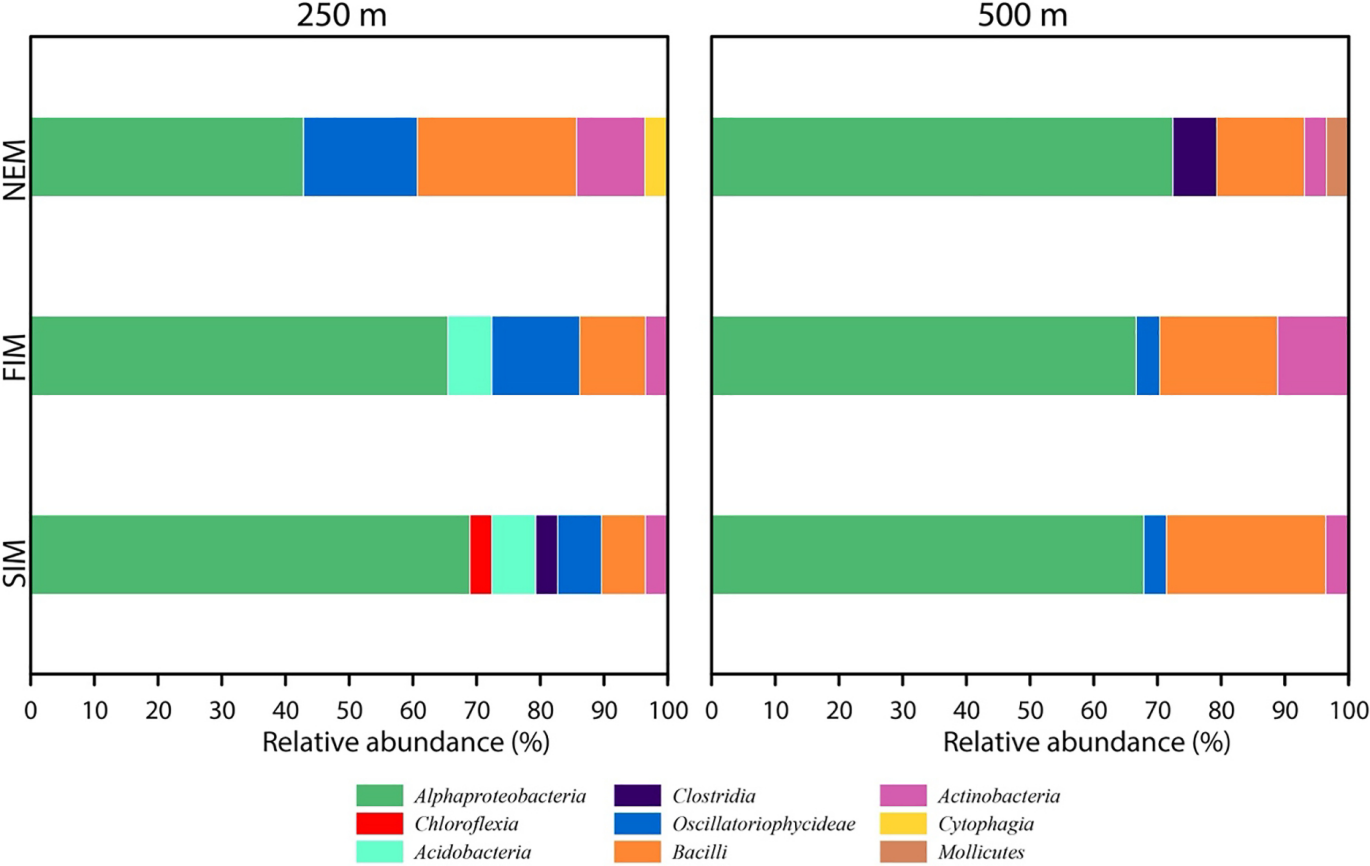
Season-wise distribution of *nosZ* bacterial classes at 250 and 500‍ ‍m at the ASTS location. See Fig. 1 for abbreviations.

The seasonal distribution patterns of *nosZ* gene sequences at the class level from 250 and 500‍ ‍m showed that the maximum number of sequences was affiliated with the class‍ ‍*Alphaproteobacteria*. At 250 m, *Alphaproteobacteria*, *Oscillatoriophycideae*, *Bacilli*, and *Actinobacteria* were detected during all three seasons. *Acidobacteria* was found during the SIM and FIM, *Chloroflexia* only during the SIM, and *Clostridia* and *Cytophagia* only during the NEM. The maximum number of classes was detected in the SIM (7), followed by the FIM (5) and NEM (5) (Fig. 4). At 500 m, *Alphaproteobacteria* was predominant, followed by *Bacilli*, *Actinobacteria*, and *Oscillatoriophycideae*. *Clostridia* and *Mollicutes* were the other classes present at small percentages. No significant variations among classes were observed between seasons, except for *Clostridia* and *Mollicutes*, which were only found during the NEM (Fig. 4). The number of classes detected at 250‍ ‍m (8) was higher than that detected at 500‍ ‍m (6).

### Abundance of the *nosZ* gene

**Fig. 5.**
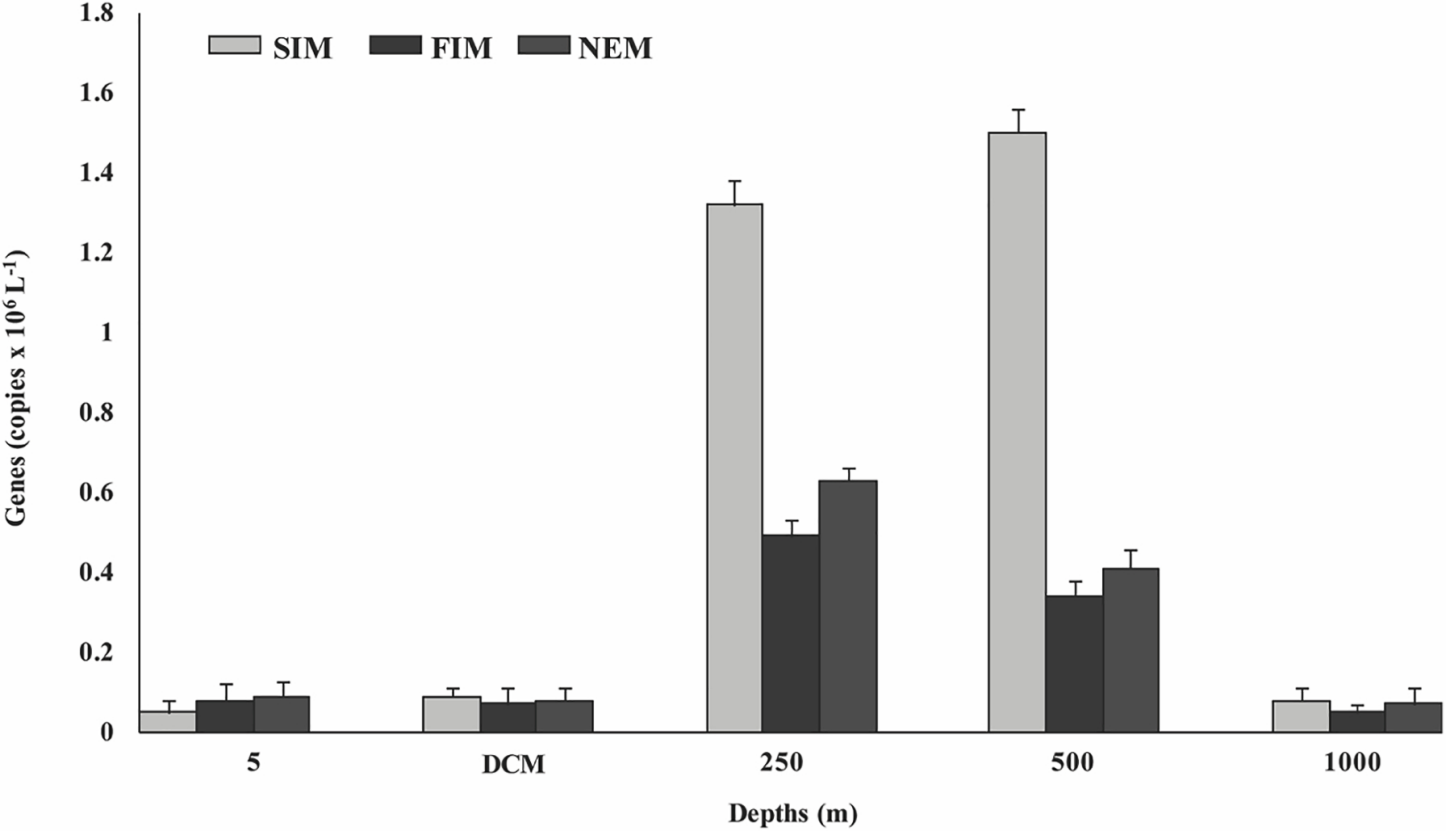
Season- and depth-wise distribution of *nosZ* gene copy numbers at the ASTS location. Depths are 5 m, deep chlo­rophyll maximum (DCM), 250 m, 500 m, and 1,000 m. See Fig. 1 for abbreviations.

Standard curves for the *nosZ* gene were plotted and qPCR efficiency (81.93%) was calculated. The efficiency reading was used as a reference to calculate the concentrations of the gene in environmental DNA samples. At surface and DCM depths, the copy numbers of the *nosZ* gene were negligible, ranging between 0.05 and 0.09×10^6^ copies L^–1^ and between 0.07 and 0.09×10^6^ copies L^–1^, respectively. At 250‍ ‍m, the abundance of *nosZ* was the highest during the SIM (1.32×10^6^ copies L^–1^), followed by the NEM (0.63×10^6^ copies L^–1^) and FIM (0.49×10^6^ copies L^–1^). At 500 m, the abundance of the *nosZ* gene was the highest during the SIM (1.50×10^6^ copies L^–1^), followed by the NEM (0.63×10^6^ copies L^–1^) and FIM (0.41×10^6^ copies L^–1^). Overall, the highest copy number of the *nosZ* gene was detected at 500‍ ‍m during the SIM (1.50×10^6^ copies L^–1^). The abundance of the *nosZ* gene at 1,000‍ ‍m varied from 0.05 to 0.08×10^6^ copies L^–1^. Fig. 5 shows the seasonal abundance and distribution of *nosZ* genes.

### Effects of environmental parameters on OTUs and copy numbers

**Fig. 6.**
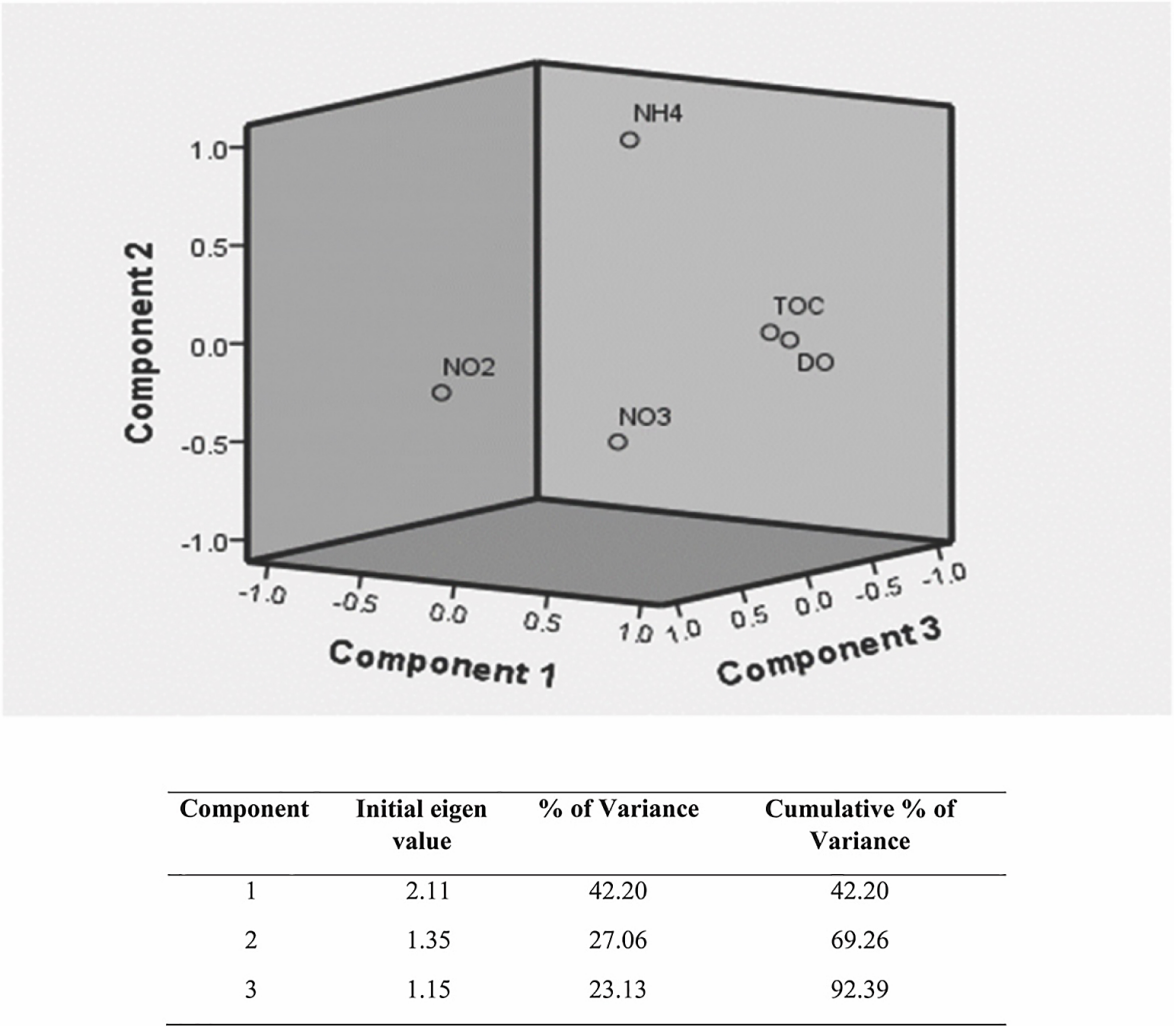
Principal component ana­lysis (PCA) plot of the rotated space with DO, TOC, NO_2_^–^ NO_3_^–^, NH_4_^+^, OTU, and copy numbers. See Fig. 1 for abbreviations.

During the SIM, DO (r=–0.701; *P*<0.01), nitrite (r=–‍0.985; *P*<0.001), and TOC (r=–0.593; *P*<0.05) negatively correlated with *nosZ* gene copy numbers, whereas ammonia (r=0.682; *P*<0.01) showed a positive correlation. Environmental parameters did not correlate with the abundance of the *nosZ* gene during the FIM, except for nitrite (r=–0.576; *P*<0.05), which showed a negative correlation. DO (r=–‍0.747; *P*<0.01) negatively correlated with the distribution of the *nosZ* gene during the NEM, while this correlation was positive for nitrite (r=0.827; *P*<0.001) (Table 2). A one-way ANOVA confirmed that physicochemical parameters, OTUs, and copy numbers significantly varied between depths (Table S1). Tukey’s post-hoc tests showed significant differences in DO, TOC, NO_3_^–^, and copy numbers between 250 and 500‍ ‍m (Table S2) during all three seasons. PCA revealed three principal components with eigenvalues >1, which explained 92.39% of all variations in physicochemical parameters. The first component (PC1) explained a total variance of 42.20% and reflected a strong gradient caused by DO (0.943) and TOC (0.916). The second component (PC2) accounted for 27.06% of variance and was reflected by NH_4_^+^ (0.958). The third component (PC3) explained 23.13% of variance and included NO_2_^–^ (0.947) (Fig. 6). Multiple regression ana­lyses showed that OTUs and copy numbers were influenced by DO, TOC, NO_2_^–^, and NO_3_^–^, while copy numbers were influenced by DO, TOC, and NH_4_^+^ (Table S3).

### Richness, OTUs, and rarefaction curves

**Fig. 7.**
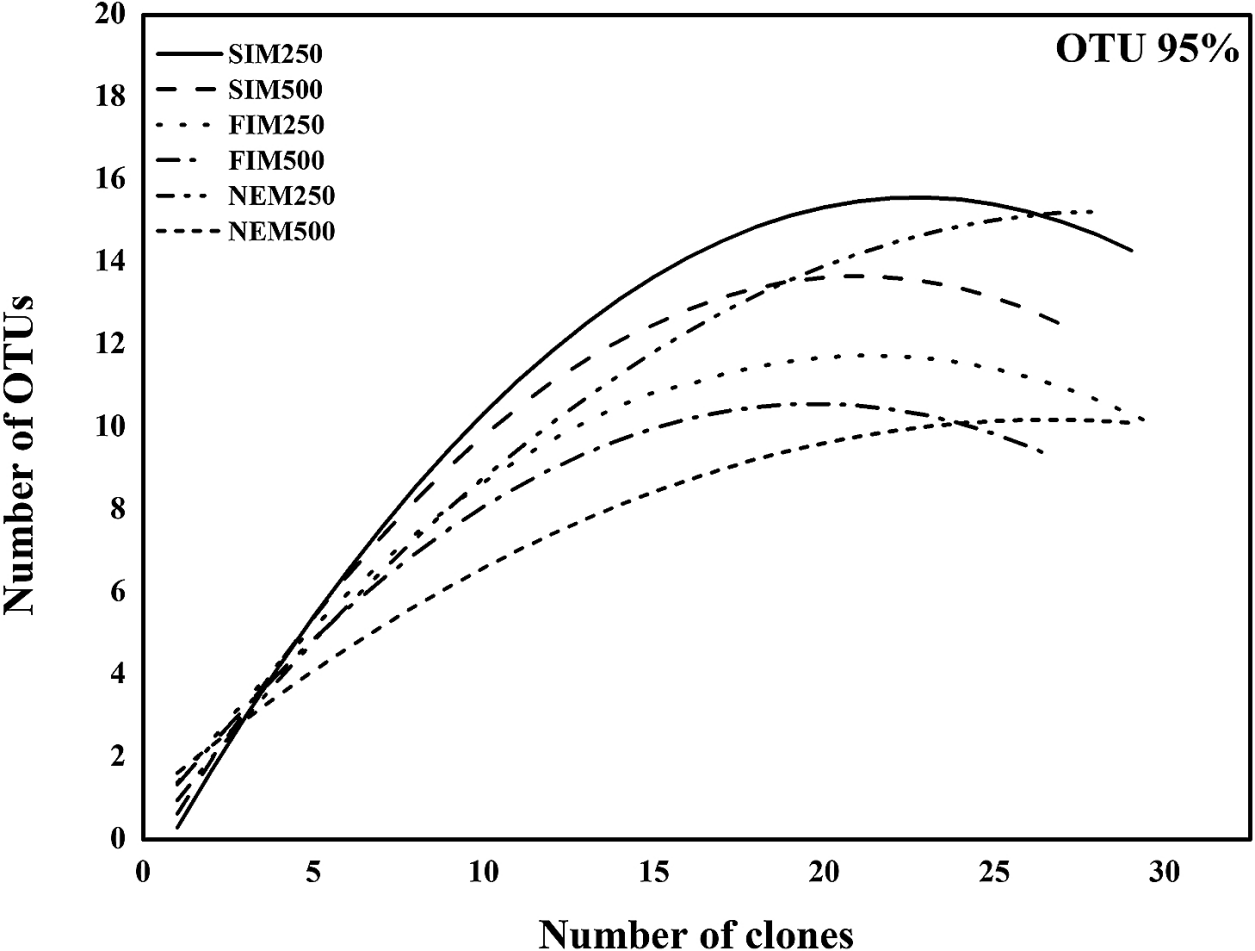
Rarefaction curves of *nosZ* gene sequences at the ASTS location. See Fig. 1 for abbreviations.

Shannon’s and Simpson’s diversity indices revealed that the diversity of *nosZ* denitrifiers was the highest during the SIM, followed by the FIM and NEM. Diversity was the highest at 250‍ ‍m during all sampling seasons. The non-parametric estimators, Chao 1 and ACE showed a higher number of OTUs at 250‍ ‍m during all seasons. The SIM (250‍ ‍m) and NEM (250 m) had the highest number of OTUs (Table 3). A rarefaction curve was plotted based on the number of clones and OTUs to investigate the relationship between sampling efforts and diversity. The saturation of the rarefaction curve indicated that the sampling effort sufficiently covered *nosZ*-denitrifying bacteria (Fig. 7).

## Discussion

In the highly productive surface waters (0–150 m) of the AS-OMZ, the *in situ* oxidation and rapid decomposition of organic matter leads to the near-exhaustion of DO at intermediate depths (~200–1,200‍ ‍m column) triggering intense denitrification (Naqvi *et al.*, 1990), which, in turn, causes the copious efflux of N_2_O into the atmosphere (Law and Owens, 1990; Arévalo-Martínez *et al.*, 2015). Although present in small numbers, *nosZ* denitrifiers are diverse (Jones *et al.*, 2013) and play a critical role in nitrous oxide production. However, most studies conducted on *nosZ* bacteria associated with denitrification are performed on cultures and do not represent the total microbial community. Therefore, detection and characterization using a metagenomic approach are important for recognizing the *nosZ* bacterial community, which is critical in denitrification.

Although mostly detected in the soil ecosystem (Sanford *et al.*, 2012), recent metagenomic sequencing studies identified Clades I and II *nosZ* genes in regions associated with the OMZ of the Eastern Tropical Pacific, the AS, and in oxygenated surface waters of the Arctic and Southern Oceans (Jayakumar *et al.*, 2018). The majority of studies that attempted to characterize *nosZ* gene diversity using DNA-based PCR approaches mainly focused on Clade I *nosZ* because the high diversity of Clade II *nosZ* makes it challenging to design a universal primer set that effectively amplifies all *nosZ* genes in this clade. The primer pair (nosZ1F/nosZ1R; Henry *et al.*, 2006) used in the present study was suitable for both marine and terrestrial *nosZ* sequences. This primer amplifies a shorter region of 259 bps than those developed by others (1,100 bp; Scala and Kerkhof, 1999) for marine targets, and, thus, was more suitable for the present study. Although these primers may not efficiently amplify and cover the sequence divergence of Clade II *nosZ* sequences (Jayakumar *et al.*, 2018), previous studies using microarrays showed that they amplified some of these sequences (Jayakumar *et al.*, 2018). *Rhodobacteraceaea* affiliated with Clade II *nosZ* genes detected in the present study was identified in the anoxic section of a wastewater treatment plant using Clade II *nosZ* gene-specific primers (DaeHyun, D.K., *et al.*, 2019 Development of group-specific *nosZ* quantification method targeting active nitrous oxide reducing population in complex environmental samples. *bioRxiv* https://doi.org/10.1101/710483). Nevertheless, we acknowledge that the PCR primers used in the present study may have been biased towards the detection of Clade I *nosZ* genes and, thus, we may have underestimated the real abundance of *nosZ* genes in our samples. Nevertheless, the present results indicate that marine *nosZ* denitrifiers (Clade I) inhabit core AS-OMZ depths and play an equal and significant role at the ASTS, leading to a high percentage of fixed nitrogen loss.

### The *nosZ* phylogeny

The absence of *nosZ*-denitrifying bacteria from the surface, DCM, and 1,000 m, and its invariable presence in samples from 250 and 500‍ ‍m during all three seasons is consistent with previous findings from the AS-OMZ (Bandekar *et al.*, 2018b). As the level of oxygen falls below the detection limit (Devol, 1978), conditions become favorable for denitrification (Codispoti *et al.*, 2005). The persistence of low oxygen levels in the core of the OMZ may be a factor limiting *nosZ*-denitrifying bacteria to depths of 250 and 500‍ ‍m (Castro-González *et al.*, 2015).

The AS-OMZ with intense upwelling and the low exchange of intermediate waters provides a high redox environment for the growth and multiplication of denitrifying bacteria. The present results demonstrated that *nosZ* denitrifiers inhabited the core of the AS-OMZ with DO levels fluctuating between 0.76–11‍ ‍μmol L^–1^, similar to findings from the Colombian Pacific Bay (CPB; Castro-González *et al.*, 2018). All *nosZ* sequences obtained from the ASTS were affiliated with the phylum Proteobacteria, as was also reported by Gomes *et al.* (2018), Divya *et al.* (2017), and Wang *et al.* (2017). Proteobacteria have been identified as the dominant phylum and play a significant role in denitrification in the AS-OMZ (Bandekar *et al.*, 2018a; Gomes *et al.*, 2018). OTUs obtained from the ASTS aligned into three‍ ‍classes: *Alphaproteobacteria*, *Betaproteobacteria*, and *Gammaproteobacteria*, which is consistent with findings from the OMZ of the Subtropical Deep Reservoir (Zheng *et al.*, 2014) and sediments of the AS (Amberkar *et al.*, 2021). *nosZ* denitrifiers from the present study were predominant within the class *Alphaproteobacteria*, which is in accordance with the findings of Wyman *et al.* (2013) from the AS.

*The nosZ* denitrifiers identified in the present study were dominated by phylotypes affiliated to *Pseudomonas*, *Rhodopseudomonas*, *Bradyrhizobium*, and *Alphaproteobacteria*. This is similar to the findings of Gomes *et al.* (2018) and Castro-González *et al.* (2018). It is important to note that *Bradyrhizobium*, an aerobic anoxygenic phototrophic bacterium typically reported in oxic waters (Hu *et al.*, 2006), was found at core OMZ depths in the present study. Few OTUs from the ASTS were homologous with the novel sequence of *Nisaea denitrificans* (class *Alphaproteobacteria*) isolated from the Mediterranean Sea, which is potentially involved in denitrification (Urios *et al.*, 2008).

Sequences from the ASTS showed similarity to the *nosZ* gene isolates of *Achromobacter* detected from the OMZ of the CPB (Castro-González *et al.*, 2018), *Herbaspirillum* identified in anaerobic wastewater treatment plants (Hou *et al.*, 2012), *Sinorhizobium* reported in the sediments of the Atlantic (Scala and Kerkhof, 1998), and *Azospirillum* found in eutrophic freshwater lakes (Wang *et al.*, 2012). We also identified *NosZ* denitrifiers affiliated with *Burkholderia*, *Alcaligenes*, and *Cupriavidus*, which were previously detected in boreal peat moss (Nie *et al.*, 2015), wastewater treatment plants (Wang *et al.*, 2015), and marsh soils (Henry *et al.*, 2006), respectively. The majority of sequences identified in the present study showed homogeneity to those reported by Gomes *et al.* (2018) from the seasonal OMZ in the AS. All of the *nosZ* denitrifiers identified in the present study were actively involved in nitrous oxide production. Some OTU sequences from this study aligned with the cultured, facultative anaerobe *Thalassobaculum* (family *Rhodospirillaceae*), the role of which remains unknown (Zhang *et al.*, 2008).

### Abundance and distribution of the *nosZ* gene

*NosZ* denitrifiers were uncommon at 5 m, DCM, and 1,000 m. Quantitative ana­lyses of the *nosZ* gene from these depths showed lower Ct values than those at core OMZ depths (250 and 500 m). PCR with a higher concentration of DNA did not yield positive amplification for any of the samples taken from depths of 5 m, DCM, or 1,000 m. Therefore, *nosZ* genes at these depths were limited and hard to detect via conventional PCR. Additionally, Staggemeier *et al.* (2015); Nagdev *et al.* (2015), and Zemtsova *et al.* (2015) reported that the sensitivity of qPCR was higher than that of conventional PCR. Melt curves, the melting temperature, and all other protocols confirmed that qPCR amplification at 5 m, DCM, and 1,000‍ ‍m was not an artefact.

Although differences were observed in abundance, the highest copy numbers during all three seasons were detected at core OMZ depths. Therefore, the oxygen concentration at these depths was the most suitable for denitrifying bacteria, indicating the perennial survival of *nosZ* denitrifiers in the AS-OMZ. The higher abundance of *nosZ* denitrifiers during the SIM may be attributed to organic carbon in the OMZ being a significant substrate that supports the existence of denitrifying and anammox bacteria (Dang *et al.*, 2010). During the SIM, bacterial communities in the AS-OMZ are sustained by slow-to-degrade dissolved organic carbon (DOC) (Ramaiah *et al.*, 2000) *i.e.*, the SIM is a transitional phase with low primary productivity (Madhupratap *et al.*, 1996) due to the persistence of oligotrophic conditions and stratification.

We herein reported a higher abundance of N_2_O-reducing bacteria from the ASTS than *nirS* denitrifiers (Bandekar *et al.*, 2018b). The *nirS* and *nosZ* genes are both assumed to be present in the genome as single-copy genes; however, there are exceptions for *nosZ* genes (Sanford *et al.*, 2012). One possible explanation for differences in abundance is that not all N_2_O-consuming bacteria contain a complete denitrification gene sequence (Sanford *et al.*, 2012). *nosZ* gene-associated bacteria lack the other steps required for conventional denitrification. In comparisons with other ecosystems, bacteria with only *nosZ* genes are over-represented in the genomes of marine bacteria (Graf *et al.*, 2014). However, the *nirS* gene, is associated with bacteria that contain a complete denitrification pathway (Graf *et al.*, 2014). Another contributing factor that may explain this difference is the specificity of PCR primers. The primers used in the present study represent a more extensive database of *nosZ* sequences (terrestrial and marine sequences), whereas the *nirS* primers used in previous studies (Bandekar *et al.*, 2018b) are potentially biased towards marine sequences (Braker *et al.*, 1998).

### Diversity and richness estimation

Stevens and Ulloa (2008) reported that DO and organic matter were important factors affecting the microbial community composition in the OMZ. The present results suggest that DO and TOC play a critical role in influencing the diversity and abundance of *nosZ* denitrifiers during different seasons. The presence of denitrifiers at core OMZ depths (as is implicit in derived diversity indices and the richness estimators ACE and Chao 1) indicated that low concentrations of oxygen, nitrite, and ammonia provided an ideal environment for the presence of *nosZ* denitrifiers (Bandekar *et al.*, 2018b). In contrast to the findings of Castro-González *et al.* (2018), the present results showed a higher diversity of *nosZ* denitrifiers at 250‍ ‍m than at 500 m. Although the sampling size in the present study was not very large, the saturation of rarefaction curves indicates that the diversity of *nosZ* denitrifiers was adequately covered.

## Conclusion

In the OMZ layers of the Arabian Sea, denitrification is a crucial pathway (Ward *et al.*, 2009; Dalsgaard *et al.*, 2012; Jain *et al.*, 2014; Bandekar *et al.*, 2018a) that has enabled the occurrence and sizable abundance of a diverse group of microbial communities, including those not taking part in the process, *per se*. The present results showed that the diversity *nosZ* denitrifiers was low and limited to core OMZ depths, suggesting that low concentrations of organic matter in OMZs not only reduce the number of available niches for microbes (Bryant *et al.*, 2012), but also unfavorably influence the denitrifying microbial community structure. While *nirS*-possessing denitrifiers control N_2_O emissions (Conrad, 1996; Wallenstein *et al.*, 2006), the higher abundance of the *nosZ* gene than *nirS* from the ASTS in the present study suggests otherwise. Furthermore, our results on hydrographic parameters indicate that the concentrations of DO and TOC‍ ‍influence the abundance and distribution of *nosZ*-denitrifying bacteria at core OMZ depths of the AS.

## Citation

Bandekar, M., Ramaiah, N., Seleyi, S. C.., Nazareth, D. R.., and Kekäläinen, J. (2023) Diversity and Quantitative Detection of Clade I Type *nosZ* Denitrifiers in the Arabian Sea Oxygen Minimum Zone. *Microbes Environ ***38**: ME22056.

https://doi.org/10.1264/jsme2.ME22056

## Supplementary Material

Supplementary Material

## Figures and Tables

**Table 1.** Spatial (depth) variations at 250 and 500‍ ‍m in different physicochemical parameters at the Arabian Sea Time Series (ASTS) location during SIM, FIM, and NEM seasons.

**Season**		**SIM**			**FIM**			**NEM**	
**Depth (m)**		**250**	**500**		**250**	**500**		**250**	**500**
Temperature (°C)		18.24±0.02	12.96±0.02		16.00±1.00	12.02±0.01		15.28±0.02	12.11±0.02
Salinity (ppt)		35.72±0.03	35.71±0.02		36.08±0.01	35.62±0.01		35.75±0.02	35.60±0.02
DO (μmol L^–1^)		11.79±0.03	4.58±0.02		3.47±0.01	0.77±0.02		1.41±0.07	1.51±0.02
pH		7.76±0.02	7.59±0.03		7.22±0.43	7.19±0.02		7.51±0.02	7.43±0.02
NO_3_^–^ (μmol L^–1^)		22.41±0.02	15.33±0.03		25.00±0.43	32.79±0.02		19.05±0.02	25.98±0.02
NO_2_^–^ (μmol L^–1^)		0.01±0.00	ND		ND	ND		2.57±0.02	0.06±0.00
PO_4_^3–^ (μmol L^–1^)		2.00±0.01	1.60±0.02		2.35±0.04	2.55±0.02		2.22±0.01	2.58±0.02
SiO_3_^2–^ (μmol L^–1^)		18.47±0.01	19.44±0.02		21.79±0.04	34.99±0.02		29.36±0.01	50.29±0.02
NH_4_^+^ (μmol L^–1^)		0.10±0.00	1.01±0.00		0.19±0.02	0.27±0.02		ND	ND
TOC (μmol L^–1^)		54.23±0.21	47.80±0.26		52.43±0.03	43.36±0.01		45.30±0.02	38.19±0.02

The Spring intermonsoon (SIM), Fall intermonsoon (FIM), and Northeast monsoon (NEM), Dissolved oxygen (DO), Nitrate (NO_3_^–^), Nitrite (NO_2_^–^), Phosphate (PO_4_^3–^), Silicate (SiO_3_^2–^), Ammonia (NH_4_^+^), Total organic carbon (TOC), and ND=not detected

**Table 2.** Spearman’s correlation coefficients (r-values) between physicochemical parameters and *nosZ* gene copy numbers at the ASTS location during the SIM, FIM, and NEM. See Table 1 for abbreviations.

**Location**	**Season**	**Genes**	**Variable**	**r**	***P*** ** value**
**ASTS**	**SIM**	***nosZ***	Depth	0.352	>0.05
			DO	–**0.701**	**0.01**
			NO_3_^–^	0.223	>0.05
			NO_2_^–^	–**0.985**	**0.001**
			NH_4_^+^	**0.682**	**0.01**
			TOC	–**0.593**	**0.05**
	**FIM**	***nosZ***	Depth	–0.356	>0.05
			DO	–0.018	>0.05
			NO_3_^–^	–0.192	>0.05
			NO_2_^–^	–**0.576**	**0.05**
			NH_4_^+^	–0.059	>0.05
			TOC	0.083	>0.05
	**NEM**	***nosZ***	Depth	0.318	>0.05
			DO	–**0.747**	**0.01**
			NO_3_^–^	0.332	>0.05
			NO_2_^–^	**0.827**	**0.001**
			NH_4_^+^	–0.483	>0.05
			TOC	–0.605	>0.05

*P*<0.05; *P*<0.01; *P*<0.001—levels of significance

**Table 3.** Diversity of *nosZ* denitrifier libraries constructed from the ASTS location during the SIM, FIM, and NEM. See Table 1 for abbreviations.

**Seasons**	**Depth (m)**	**Sequences**	**OTUs**	**Richness estimation**			
				**Shannon**	**Simpsons**	**Chao**	**ACE**
**SIM**	250	29	**15**	**3.56**	**0.03**	16.8	23.4
	500	28	13	3.49	0.01	28.3	26.28
**FIM**	250	30	**11**	**3.27**	**0.02**	85.2	107.31
	500	27	10	2.9	0.04	25	28.39
**NEM**	250	28	**15**	**2.92**	**0.04**	25.5	27.1
	500	29	11	2.27	0.09	11.33	11.69
