# Supplementary material for: Diversity and Quantitative Detection of Clade I Type nosZ Denitrifiers in the Arabian Sea Oxygen Minimum Zone
Source: Microbes Environ. 2023 Jan 26;38(1):ME22056. doi: 10.1264/jsme2.ME22056 (PMC10037096; doi:10.1264/jsme2.ME22056)
Supplement: Supplementary file 1 — Supplementary Material [file 38_22056_s1.pdf]

Table S1. One-way ANOVA analysis and significance (*p* <0.05) of variations in physicochemical parameters.

	**F value**	***P***
**DO**	45008.028	<0.0001
**NO_3^-**	3473.537	<0.0001
**NO_2^-**	44326.843	<0.0001
**NH_4^+**	3555.154	<0.0001
**TOC**	5520.673	<0.0001
**OTU**	50.100	<0.0001
**copy numbers**	2431.393	<0.0001

Dissolved oxygen (DO), Nitrate ($NO3^-$), Nitrite ($NO2^-$), Ammonia ($NH4^+$), Total organic carbon (TOC), Operational Taxonomic unit (OTU)

Table S2: Tukey's post hoc comparisons (2-sided) for physicochemical parameters, OTUs and copy numbers between depths (250 m and 500 m) within each season. See Table S1 for abbreviations.

Dependent variables	SIM 250 vs SIM 500	FIM250 vs FIM 500	NEM 250 vs NEM 500
**DO**	**<0.0001**	**<0.0001**	0.025
**TOC**	**<0.0001**	**<0.0001**	**<0.0001**
**NO_2^-**	0.657	1.00	**<0.0001**
**NO_3^-**	**<0.0001**	**<0.0001**	**<0.0001**
**NH_4^+**	**<0.0001**	**<0.0001**	1.00
**OTU**	0.214	0.817	**<0.0001**
**copy number**	**<0.0001**	**<0.0001**	**<0.0001**

The mean difference is significant at $p<0.05$ level.

Table S3: Multiple regression models predicting the association between bacteria diversity (OTU) and abundance (copy number) and principal component scores for the physico-chemical parameters (DO, TOC, $NO2^-$ $NO3^-$ and $NH4^+$). See Table S1 for abbreviations.

**Dependent variables**	**Predictor**	**Standardized Coefficient (β)**	**t-value**	**Significance (*p*)**	**R^2**	**Adjusted R^2**
**OTU**	Component 1	0.202	2.457	0.028	0.90	0.885
	Component 2	-0.050	-0.612	0.551		
	Component 3	0.928	11.292	<0.0001		
**copy number**	Component 1	0.640	6.842	<0.0001	0.87	0.851
	Component 2	0.678	7.243	<0.0001		
	Component 3	0.093	0.996	0.336		

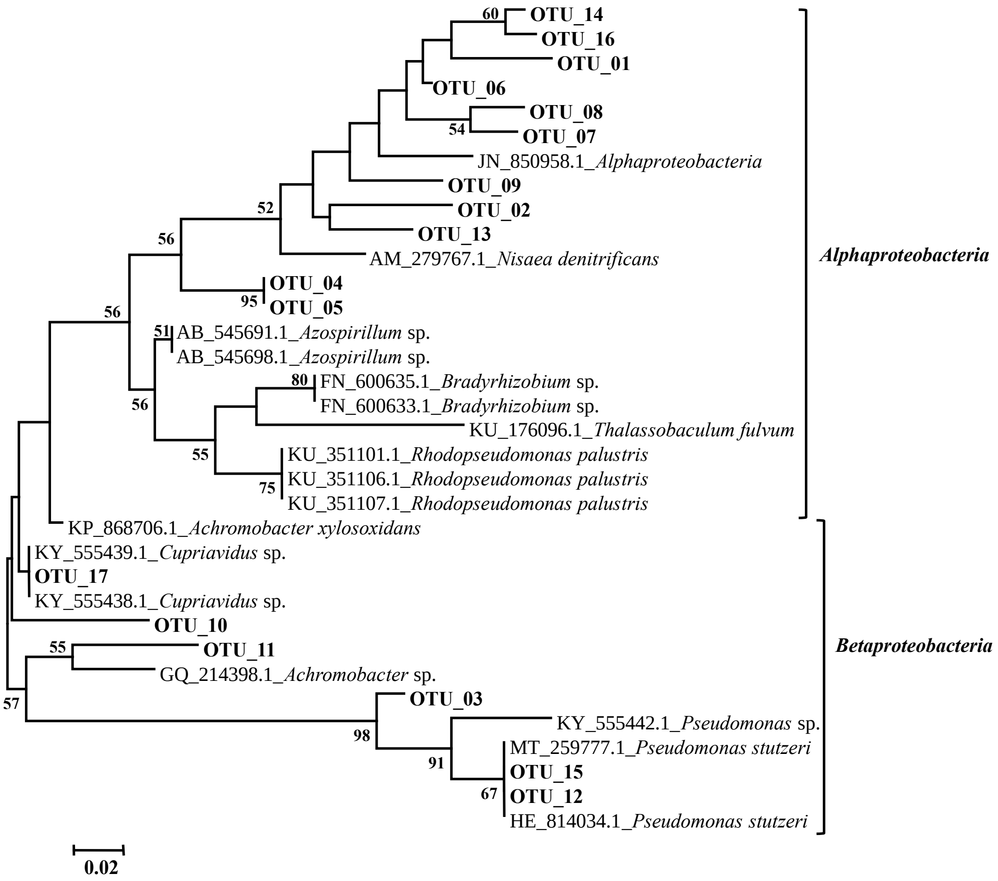

Figure S1. Phylogenetic tree constructed using Neighbor-joining method of the *nosZ* gene OTU sequences obtained from nosZ250m at the ASTS location. The sequences in bold are from the present study.

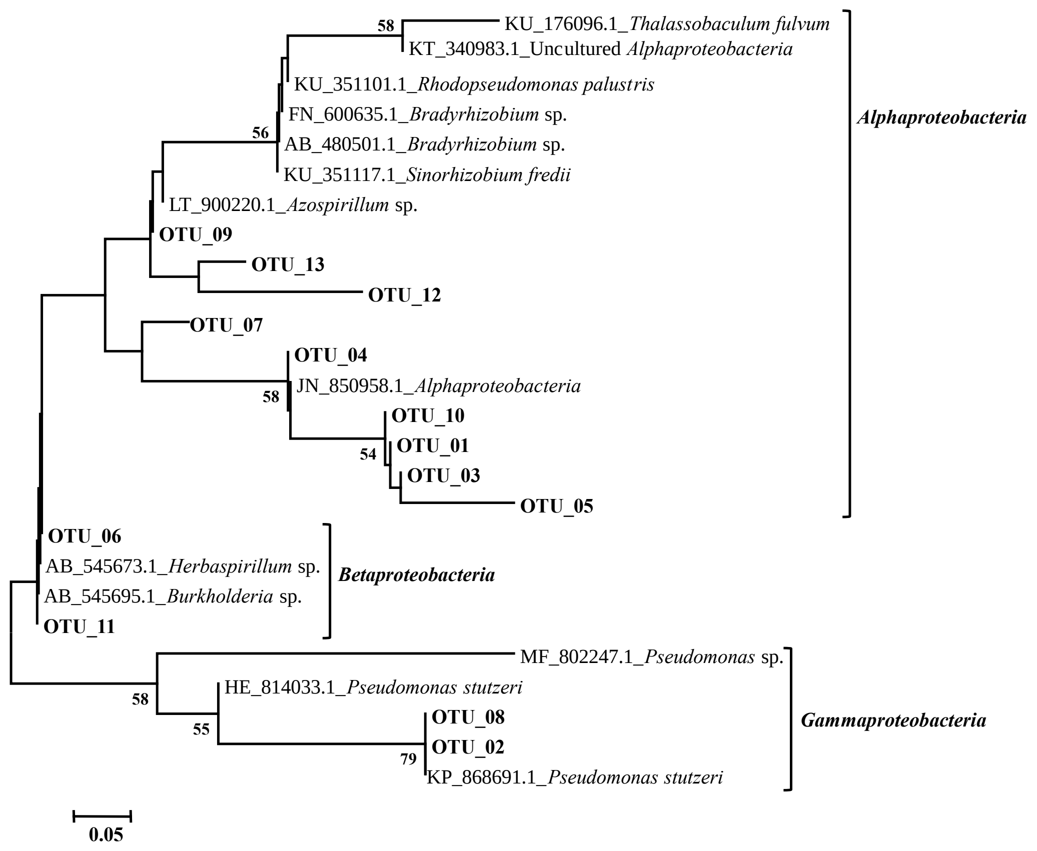

Figure S2. Phylogenetic tree constructed using Neighbor-joining method of the *nosZ* gene OTU sequences obtained from nosZ500m at the ASTS location. The sequences in bold are from the present study.
